# Endocrine-Based Treatments in Clinically-Relevant Subgroups of Hormone Receptor-Positive/HER2-Negative Metastatic Breast Cancer: Systematic Review and Meta-Analysis

**DOI:** 10.3390/cancers13061458

**Published:** 2021-03-22

**Authors:** Francesco Schettini, Mario Giuliano, Fabiola Giudici, Benedetta Conte, Pietro De Placido, Sergio Venturini, Carla Rognoni, Angelo Di Leo, Mariavittoria Locci, Guy Jerusalem, Lucia Del Mastro, Fabio Puglisi, PierFranco Conte, Michelino De Laurentiis, Lajos Pusztai, Mothaffar F. Rimawi, Rachel Schiff, Grazia Arpino, Sabino De Placido, Aleix Prat, Daniele Generali

**Affiliations:** 1 Department of Clinical Medicine and Surgery, University of Naples “Federico II”, 80131 Naples, Italy; m.giuliano@unina.it (M.G.); pietrodep91@gmail.com (P.D.P.); grazia.arpino@unina.it (G.A.); deplacid@unina.it (S.D.P.); 2 Translational Genomics and Targeted Therapies in Solid Tumors, IDIBAPS, 08036 Barcelona, Spain; bntconte@gmail.com (B.C.); alprat@clinic.cat (A.P.); 3 SOLTI Breast Cancer Research Group, 08008 Barcelona, Spain; 4 Unit of Biostatistics, Department of Cardiac, Thoracic, Vascular Sciences and Public Health, University of Padova, 35122 Padova, Italy; fgiudici@units.it; 5 Breast Unit, IRCCS Ospedale Policlinico San Martino, 16132 Genoa, Italy; lucia.delmastro@hsanmartino.it; 6 Department of Management, University of Turin, 10124 Turin, Italy; sergio.venturini@unibocconi.it; 7 Centre for Research on Health and Social Care Management (CERGAS), SDA Bocconi School of Management, 20136 Milan, Italy; carla.rognoni@unibocconi.it; 8 “Sandro Pitigliani” Medical Oncology Department, Hospital of Prato, 59100 Prato, Italy; angelo.dileo@uslcentro.toscana.it; 9 Department of Neuroscience, Reproductive Medicine and Odontostomatological Sciences, University of Naples Federico II, 80131 Naples, Italy; mariavittorialocci@virgilio.it; 10 Medical Oncology Department, Centre Hospitalier Universitaire de Liège and Liège University, 4000 Liège, Belgium; g.jerusalem@chu.ulg.ac.be; 11 Department of Internal Medicine and Medical Specialties (DIMI), School of Medicine, University of Genoa, 16132 Genoa, Italy; 12 Department of Medicine, University of Udine, 33100 Udine, Italy; fabio.puglisi@uniud.it; 13 IRCCS Centro di Riferimento Oncologico Aviano, National Cancer Institute, 33081 Aviano, Italy; 14 Department of Surgery, Oncology and Gastroenterology, University of Padova, 35122 Padova, Italy; pierfranco.conte@unipd.it; 15 Division of Medical Oncology 2, Istituto Oncologico Veneto–IRCCSS, 35128 Padova, Italy; 16 Breast Oncology Unit, INT-Fondazione “G. Pascale”, 80131 Naples, Italy; m.delaurentiis@istitutotumori.na.it; 17 Department of Medicine, Yale University School of Medicine, New Haven, CT 06510, USA; lajos.pusztai@yale.edu; 18 Dan L Duncan Comprehensive Cancer Center, Baylor College of Medicine, Houston, TX 77030, USA; rimawi@bcm.edu (M.F.R.); rschiff@bcm.edu (R.S.); 19 Department of Medicine, Baylor College of Medicine, Houston, TX 77030, USA; 20 Department of Molecular and Cellular Biology, Baylor College of Medicine, Houston, TX 77030, USA; 21 Department of Medical Oncology, Hospital Clínic, 08036 Barcelona, Spain; 22 Department of Medical, Surgery and Health Sciences, University of Trieste, 34127 Trieste, Italy; 23 Breast Cancer Unit, Azienda Socio Sanitaria Territoriale di Cremona, 26100 Cremona, Italy

**Keywords:** endocrine therapy, hormone receptor, metastatic breast cancer, meta-analysis, systematic review

## Abstract

**Simple Summary:**

Hormone receptor-positive (HR+)/HER2-negative is the most frequent subgroup of metastatic breast cancer (MBC). Important therapeutic advances in the treatment of this tumor type have been observed in the last 20 years, with the approval of numerous endocrine therapies (ET) with or without target therapies (TT). To improve our current knowledge and support clinical decision-making, we conducted a systematic literature and meta-analysis focused on the most relevant/promising first-/second-line ET ± TT of the last 20 years. We observed that CDK4/6-inhibitors(i) + ET were the most effective regimens. At the same time, mTORi-based combinations proved to be a valid therapeutic option in endocrine-resistant tumors, as well as PI3Ki + ET in PIK3CA-mutant patients. Single agent ET might still be a valuable upfront treatment in endocrine sensitive and non-visceral disease.

**Abstract:**

A precise assessment of the efficacy of first-/second-line endocrine therapies (ET) ± target therapies (TT) in clinically-relevant subgroups of hormone receptor-positive (HR+)/HER2-negative metastatic breast cancer (MBC) has not yet been conducted. To improve our current knowledge and support clinical decision-making, we thus conducted a systematic literature search to identify all first-/second-line phase II/III randomized clinical trials (RCT) of currently approved or most promising ET ± TT. Then, we performed a meta-analysis to assess progression-free (PFS) and/or overall survival (OS) benefit in several clinically-relevant prespecified subgroups. Thirty-five RCT were included (17,595 patients). Pooled results show significant reductions in the risk of relapse or death of 26–41% and 12–27%, respectively, depending on the clinical subgroup. Combination strategies proved to be more effective than single-agent ET (PFS hazard ratio (HR) range for combinations: 0.60–0.65 vs. HR range for single agent ET: 0.59–1.37; OS HR range for combinations: 0.74–0.87 vs. HR range for single agent ET: 0.68–0.98), with CDK4/6-inhibitors(i) + ET being the most effective regimen. Single agent ET showed comparable efficacy with ET+TT combinations in non-visceral (*p* = 0.63) and endocrine sensitive disease (*p* = 0.79), while mTORi-based combinations proved to be a valid therapeutic option in endocrine-resistant tumors, as well as PI3Ki + ET in PIK3CA-mutant tumors. These results strengthen international treatment guidelines and can aid therapeutic decision-making.

## 1. Introduction

About 60–75% of breast tumors are hormone receptor-positive (HR+) and do not present with HER2 overexpression or amplification (HER2−) [1]. Following the introduction of multiple novel therapeutic agents into clinical practice, HR+/HER2− metastatic breast cancer (MBC) has become a more chronic and manageable disease, albeit still incurable [2,3,4,5,6,7,8,9,10,11,12,13]. Indeed, death rates decreased by an average of 1.8% annually between 2007 and 2016 [14], and a recent meta-analysis reported a median survival increase from 32 months in 1990 (95% confidence interval (CI): 23–43) to 57 (95% CI: 37–87) months [15]. Current international guidelines recommend treating HR+/HER2− MBC by administering endocrine treatment (ET), combined or not with TT, for as long as possible before changing to chemotherapy, except in the case of “visceral crisis” [16,17,18]. In fact, we previously demonstrated the absence of significant difference between ET + TT and chemotherapy and the clear superiority of ET combined with novel CDK4/6-inhibitors (CDK4/6i) or PIK3CA/mTOR axis inhibitors compared to standard ET, in terms of both response rates and progression-free survival (PFS) [19]. However, previous network meta-analyses comparing all first-/second-line treatment options [19,20,21,22] were unable to evaluate the efficacy of these novel treatment strategies in specific clinical subsets (endocrine-resistant or endocrine-sensitive patients, patients with visceral or bone-only disease, pre-menopausal patients, etc.) and did not provide overall survival (OS) data. Therefore, to support current treatment guidelines and aid therapeutic decision-making in more specific tumor subsets, we performed a meta-analysis with preplanned subgroup analyses and meta-regression to evaluate the benefit of all currently available and most promising first-/second-line ET-based treatments in clinically relevant subgroups of HR+/HER2− MBC.

## 2. Materials and Methods

### 2.1. Search Strategy and Selection Criteria

After a systematic review of the literature [19] (details in the Text S1), we selected all phase II/III RCT published between January 2000 and December 2019 that compared the effect of ET and/or ET combined with the most relevant TT in the first-/second-line treatment of HR+/HER2− MBC. The following TT were selected: CDK4/6-inhibitors (palbociclib, ribociclib and abemaciclib), mTOR-inhibitors (mTORi; everolimus and temsirolimus), PI3K-inhibitors (PI3Ki; buparlisib, pictilisib and alpelisib), AKT-inhibitors (AKTi; capivasertib) and histone deacetylase inhibitors (HDACi; entinostat). These drugs were selected because they: (i) were already approved (CDK4/6i, everolimus and alpelisib); or (ii) had shown promising results in phase II trials and were under further investigation at the time of the literature review (capivasertib and entinostat); or (iii) were of the same molecular class as the approved TT included in our study (everolimus and alpelisib) but were not approved because of drug-specific limitations (temsirolimus, pictilisib and buparlisib). As ET, we evaluated only currently approved drugs, namely tamoxifen, aromatase inhibitors (AI; anastrozole, letrozole and exemestane), progestins (megestrol acetate) and fulvestrant.

### 2.2. Data Extraction

Details of the study design, patient characteristics, current and previous treatment were extracted from each paper, together with hazard ratios and associated 95% confidence intervals for PFS, time-to-progression (TTP) and OS, when reported. These data had to be publicly available or computable from published paper/abstracts, otherwise studies were excluded. TTP was considered when PFS was not available, as reported elsewhere [19,21].

### 2.3. Study Endpoints

The primary endpoint was the pooled PFS/TTP and the secondary endpoint was the pooled OS estimated in prespecified clinically relevant subgroups of HR+/HER2− MBC when comparing all new ET, ET + TT or ET combinations versus the previous standard ET. We then used subgroup analysis and meta-regression to explore the effect on PFS/TTP and OS of the different treatment strategies and drug classes considered in prespecified clinically-relevant subgroups. The subgroups were the following: visceral, non-visceral and bone-only disease, PIK3CA-mutant/non-mutant tumors, endocrine-sensitive/-resistant tumors, primary and secondary endocrine-resistance and pre/peri- and postmenopause. Endocrine sensitivity status was defined according to ESO-ESMO breast cancer treatment guidelines (Text S1) [18]. All patients enrolled in RCT that investigated metastatic endocrine treatment lines after the first-line were considered to be affected by endocrine resistance. A distinction between primary or secondary resistance was not always feasible. We specifically selected the above-mentioned subgroups because we believed they were at the same time: (1) useful in the daily clinical practice; and (2) mostly retrievable from published RCT, or at least part of them.

### 2.4. Data Analysis

We generated pooled HRs using the inverse-variance weighting method. As we expected heterogeneity among studies, we selected a priori the DerSimonian and Laird random effect model [23]. Pooled data are reported in forest plots. Statistical significance was set at *p* < 0.05. All tests were two-sided. Inter-study heterogeneity was assessed by visual inspection of the forest plots and the I^2^ statistic [24]. We assessed publication bias and small study effects with funnel plots and the Egger’s test [25]. Subgroup analyses were performed if at least 3 studies comparing 2 different combinations of therapy or 2 different drug classes were available. Meta-regression was performed when at least 10 studies were available. Statistical analyses were performed using R software (R Foundation for Statistical Computing, Vienna, Austria) version 3.5.0, package meta (https://www.r-project.org (accessed on May 2018)). The risk of bias for each trial was assessed according to the criteria outlined in the Cochrane Handbook for Systematic Reviews of Interventions (available at: https://training.cochrane.org/cochrane-handbook-systematic-reviews-interventions#how-to-access (accessed on July 2019)). Internal validity of eligible studies was assessed according to the Cochrane Collaboration’s Risk of Bias tool in Review Manager (software available at: https://training.cochrane.org/online-learning/core-software-cochrane-reviews/revman/reasons-downloading-revman-5 (accessed on February 2019)). The project is registered in the Open Science Framework online repository (https://osf.io; doi: 10.17605/OSF.IO/79D4U, accessed on January 2020).

## 3. Results

### 3.1. Study Characteristics

**Figure 1:**
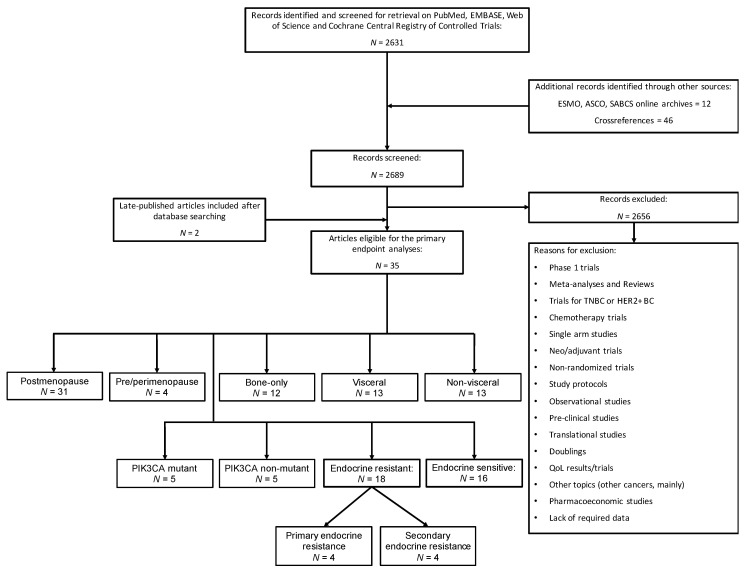
PRISMA diagram.

Based on these criteria, we identified 35 randomized controlled trials (RCT) for a total of 17,595 patients [3,4,5,6,7,8,9,10,26,27,28,29,30,31,32,33,34,35,36,37,38,39,40,41,42,43,44,45,46,47,48,49,50,51,52]. Details of the study selection are reported in the PRISMA diagram (Figure 1).

The median follow-up for the studies included was 18.8 (interquartile range (IQR): 13.0–24.2, min–max range: 4.2–87.6) months, all 35 studies were multicenter, 27 (77.1%) were phase III trials, 7 (20.0%) were phase II and 1 (2.9%) was a phase II/III trial. Sixteen studies (45.7%) included only the first-line setting, eighteen (51.4%) enrolled patients in first-line or more advanced and one trial was set in second-line or more (2.9%). Two (5.7%) studies enrolled only premenopausal patients, two (5.7%) enrolled pre- and postmenopausal patients, and thirty-one (88.6%) only enrolled postmenopausal patients. Study characteristics are detailed in Table S1. The studies were further regrouped according to treatment strategy (studies of combination treatments (ET + TT or ET combinations) compared to single agent ET vs. studies of single agent ET) and drug class (studies evaluating CDK4/6i-, mTORi-, PI3Ki-, HDCAi- and AKTi-based combinations with ET vs. single agent ET; SERD (only fulvestrant) vs. AI or SERM (only tamoxifen); SERD + AI vs. SERD; SERD + AI vs. AI and AI vs. tamoxifen).

### 3.2. Pooled Estimates in Clinical Subsets

**Figure 2:**
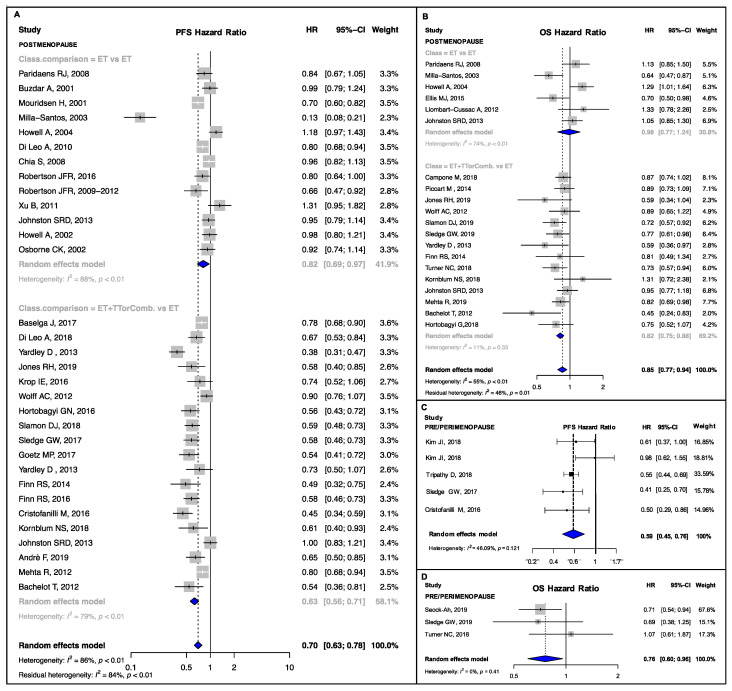
PFS and OS pooled results according to menopausal status: PFS (**A**) and OS (**B**) pooled result for the postmenopausal subgroup, overall and according to treatment strategy; and PFS (**C**) and OS (**D**) pooled result for the pre/perimenopausal subgroup. PFS, progression-free survival; OS, overall survival; ET vs. ET, studies comparing single endocrine agents therapies; ET + TTorComb. vs. ET, studies comparing endocrine therapies + target therapies or endocrine therapies combinations against single agent endocrine treatments; HR, hazard ratio; 95% CI, 95% confidence interval.

**Figure 3:**
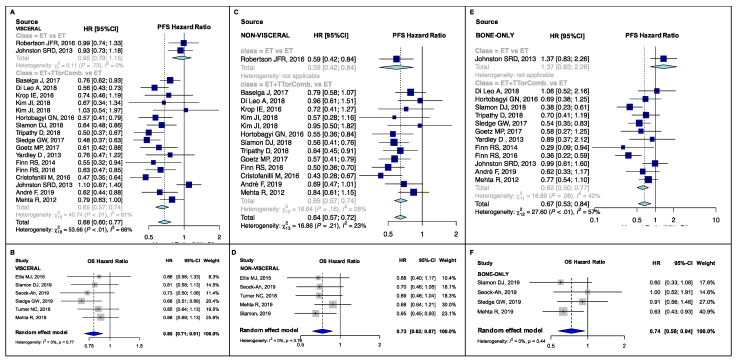
PFS and OS pooled results according to metastatic distribution: PFS pooled results for visceral (**A**), non-visceral (**C**) and bone-only disease (**E**) subgroups, overall and according to treatment strategy; and OS pooled results for visceral (**B**), non-visceral (**D**) and bone-only disease (**F**) subgroups. PFS, progression-free survival; OS, overall survival; ET vs. ET, studies comparing single endocrine agents therapies; ET + TT or Comb. vs. ET, studies comparing endocrine therapies + target therapies or endocrine therapies combinations against single agent endocrine treatments; HR, hazard ratio; 95% CI, 95% confidence interval.

**Figure 4:**
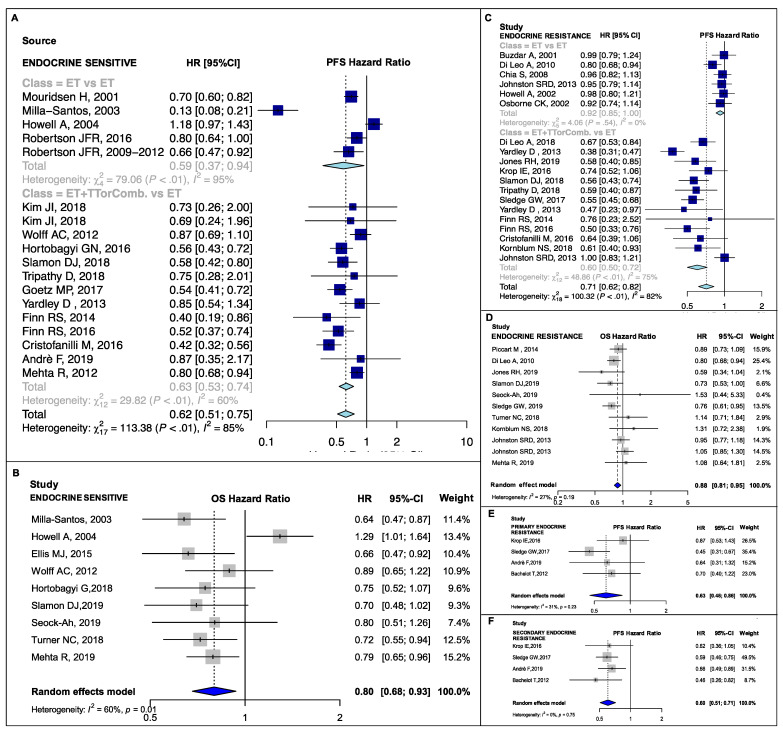
PFS and OS pooled results according to endocrine sensitiveness: PFS pooled result for the endocrine sensitive (**A**) and resistant (**C**) subgroups; OS pooled result for the endocrine sensitive (**B**) and endocrine resistant (**D**) subgroups; and PFS pooled result for the primary endocrine resistant (**E**) and secondary endocrine resistant (**F**) subgroups; PFS, progression-free survival; OS, overall survival; ET vs. ET, studies comparing single agents endocrine therapies; ET+TT or Comb. vs. ET, studies comparing endocrine therapies + target therapies or endocrine therapies combinations against single agent endocrine treatments; HR, hazard ratio; 95% CI, 95% confidence interval.

**Figure 5:**
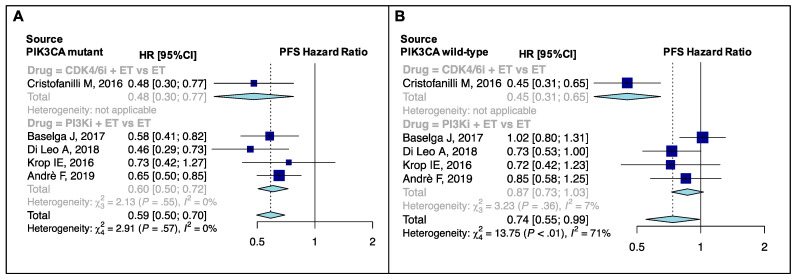
PFS pooled results according to PIK3CA mutational status: PFS pooled results for PIK3CA-mutant (**A**) and non-mutant (**B**) patients, overall and according to drug classes. PFS, progression-free survival; ET, endocrine therapy; HR, hazard ratio; 95% CI, 95% confidence interval. PI3Ki, phosphatidylinositol 3-kinases inhibitors; CDK4/6i, Cyclin-dependent kinases 4/6 inhibitors.

New ET, ET + TT and ET combinations developed in the last two decades significantly improved PFS compared to the previous standard ET (Hazard Ratio (HR) range: 0.59–0.78, *p*-value range: <0.001–0.042; I^2^ range: 0.00–86.20%, p_heterogeneity_ (p_H_) range: <0.001–0.75) (Figure 2A,C, Figure 3A,C,E, Figure 4A,C,E,F and Figure 5A,B). Heterogeneity was high in the endocrine-sensitive/resistant and postmenopausal subgroups (I^2^: 85.4%, 82.10% and 86.2%, respectively; p_H_ < 0.001 for all).

A significant pooled OS improvement was also observed in almost all subgroups (HR range: 0.73–0.88, *p*-value range: <0.001–0.02; I^2^ range: 0.00–59.9%, p_H_ range: 0.003–0.77) except in the PIK3CA-mutant and non-mutant groups (p_subgroup_ (p_sub_) = 0.08 and p_sub_ = 0.94, respectively) (Figure 2B,D, Figure 3B,D,F and Figure 4B,D). No high heterogeneity or publication bias was observed (Figure S1), except for the PFS endpoint in postmenopausal setting (Egger’s test *p* = 0.009). The results are detailed in Table 1.

### 3.3. Subgroup Analyses and Meta-Regressions: Combination Studies versus Single Agent Studies

In postmenopausal patients, the subgroup analysis showed that PFS was significantly better in studies comparing ET+TT or ET combinations than in RCT of single agent ET ((HR: 0.63, 95% CI: 0.56–0.71, I^2^: 78.8%) vs. (HR: 0.82, 95% CI: 0.69–0.97, I^2^: 87.9%), p_sub_ = 0.01, Figure 2A); this result was also confirmed by the meta-regression analysis (β meta-regression_(m)_: −0.26, p_m_ = 0.01). The result was significant also in terms of OS ((HR: 0.82, 95% CI: 0.75–0.88, I^2^: 10.8%) vs. (HR: 0.98, 95% CI: 0.77–1.24, I^2^: 74.1%), p_sub_ = 0.03, Figure 2B; β_m_: −0.16, p_m_ = 0.02)

In patients with visceral disease, the pooled PFS was significantly better in combination studies than in single agent ET studies ((HR: 0.65, 95% CI: 0.57–0.74, I^2^: 60.7%) vs. (HR: 0.95, 95% CI: 0.79–1.15, I^2^: 0.0%), p_sub_ < 0.001, Figure 3A; β_m_: −0.30, p_m_ = 0.028). No significant difference was observed in terms of OS (p_sub_ = 0.73). In non-visceral disease, there was no difference between the two study groups in terms of PFS (p_sub_ = 0.63) and OS (p_sub_ = 0.77). Nevertheless, individual PFS results for both treatment subgroups were significant ((HR: 0.65, 95% CI: 0.57–0.74, I^2^: 27.9%) vs. (HR: 0.59, 95% CI: 0.42–0.84, I^2^: not evaluable); Figure 3C). Similar to the visceral subgroup, in MBC patients with bone-only disease, PFS was better in combination treatment studies than in those receiving ET alone ((HR: 0.62, 95% CI: 0.50–0.77, I^2^: 41.8%) vs. (HR: 1.37, 95% CI: 0.83–2.26, I^2^: 0.0%), p_sub_ = 0.004, Figure 3E; β_m_: −0.79, p_m_ = 0.03). This comparison was not possible for the OS endpoint.

With respect to the endocrine-sensitive subset, neither pooled PFS nor pooled OS differed significantly in relation to treatment strategy (p_sub_ = 0.79 and p_sub_ = 0.81, respectively). Though, individual PFS results for both treatment subgroups were significant ((HR: 0.63, 95% CI: 0.53–0.74, I^2^: 59.8%) vs. (HR: 0.59, 95% CI: 0.37–0.94, I^2^: 94.9%), Figure 4A). On the contrary, in the endocrine-resistance setting, PFS was better in combination therapy studies than in single agent ET studies ((HR: 0.60, 95% CI: 0.50–0.72, I^2^: 75.4%) vs. (HR: 0.92, 95% CI: 0.85–1.00, I^2^: 0.0%), p_sub_ = 0.001, Figure 4C; β_m_: −0.43, p_m_ = 0.001). There was a similar albeit not significant (p_sub_ = 0.81) effect on OS. Progression-free survival and OS subgroup analyses for the premenopausal setting, PIK3CA-mutant and non-mutant disease and primary and secondary endocrine resistance were not feasible. We were able to conduct meta-regression for the OS endpoint only in the postmenopausal subgroup, because in the other subgroups either the number of studies was too low (<10) or subgroup analyses were not significant. PFS and OS results are detailed in Table 2 and Table S2, respectively.

### 3.4. Subgroup Analyses and Meta-Regressions: Drug Class Comparisons

Subgroup analysis of drug class revealed statistically significant differences in PFS in postmenopausal (p_sub_ = 0.01), visceral (p_sub_ < 0.001), non-visceral (p_sub_ = 0.008), bone-only (p_sub_ = 0.002), endocrine-sensitive (p_sub_ < 0.001), endocrine-resistant (p_sub_ < 0.001) and PIK3CA non-mutant (p_sub_ = 0.001) subgroups (Figure 5A,B and Figure S2). No difference was observed in the PIK3CA-mutant (p_sub_ = 0.38) subgroup.

Studies of CDK4/6i-based regimens served as reference for the meta-regressions. In the postmenopausal setting, the most pronounced beneficial effects were observed in CDK4/6i-containing group (HR: 0.55, 95% CI: 0.50–0.61) and in the group of AI compared to tamoxifen (HR: 0.44, 95% CI: 0.21–0.92). Meta-regression analysis revealed a significantly worse effect for AI vs. progestins (β_m_: 0.60, p_m_ = 0.04), SERD + AI vs. SERD (β_m_: 0.61, p_m_ = 0.03) and SERD vs. AI or tamoxifen (β_m_: 0.56, p_m_ < 0.001) study groups compared to the reference group.

In patients with visceral disease, the best pooled result was found in studies of CDK4/6i–containing regimens (HR: 0.55, 95% CI: 0.49–0.61). At meta-regression analysis, the beneficial effect was significantly less pronounced in PI3Ki-containing studies (β_m_: 0.21, p_m_ = 0.02), SERD + AI vs. AI (β_m_: 0.36, p_m_ = 0.006) and SERD vs. AI or tamoxifen (β_m_: 0.55, p_m_ = 0.001) study groups, and a significant detrimental effect was observed in the SERD + AI vs. SERD group (β_m_: 0.69, p_m_ < 0.001; Figure S2). In the non-visceral setting, CDK4/6i-containing studies showed the best pooled result (HR: 0.55, 95% CI: 0.47–0.63). Compared to the latter group, the beneficial effect was significantly lower in the PI3Ki-containing studies (β_m_: 0.36, p_m_ = 0.004), followed by the SERD+AI vs. AI group (β_m_: 0.43, p_m_ = 0.01; Figure S2). No difference was observed in the SERD vs. AI or tamoxifen group (HR: 0.59, 95% CI: 0.42–0.84; p_m_ = 0.67).

In bone-only disease, a numerically significant pooled PFS benefit was observed only in the CDK4/6i+ET vs. ET subgroup (HR: 0.50, 95% CI: 0.40–0.62). Meta-regression analysis revealed a statistically significant inferiority in studies investigating the combination of SERD + AI vs. SERD (β_m_: 0.69, p_m_ = 0.018) and SERD vs. AI or tamoxifen (β_m_: 1.01, p_m_ < 0.001%; Figure S2). In the endocrine-sensitive setting, the effect seemed to be more pronounced in the CDK4/6i+ET vs. ET group (HR: 0.52, 95% CI: 0.46–0.59; Figure S2). In the endocrine-resistance setting, the study group showing the best result was the one containing mTORi (HR: 0.47, 95% CI: 0.29–0.74). At meta-regression analysis, the beneficial effect was significantly greater in the mTORi + ET vs. ET studies (β_m_: −0.29, p_m_ = 0.016) and significantly lower in the studies comparing SERD to AI or tamoxifen (β_m_: 0.53, p_m_ < 0.001) and SERD+AI to SERD (β_m_: 0.57, p_m_ < 0.001; Figure S2) compared to the reference group.

In PIK3CA-mutant patients, the two drug classes compared, namely CDK4/6i and PI3Ki, both resulted in a better PFS vs. standard ET (HR: 0.48, 95% CI: 0.30–0.77 and HR: 0.60, 95% CI: 0.50–0.72, respectively; Figure 5A). On the contrary, in PIK3CA wild-type patients only CDK4/6i-containing studies provided a significant PFS improvement (HR: 0.45, 95% CI: 0.31–0.65; Figure 5B) compared to PI3Ki-containing studies.

Subgroup analyses for OS were not statistically significant (Table S2). PFS and OS subgroup analyses according to drug class comparisons were not feasible for the pre/perimenopausal, primary and secondary endocrine-resistance subgroups. Meta-regression could not be performed for the OS endpoint, except in the postmenopausal setting.

### 3.5. Risk of Bias Analysis

**Figure 6:**
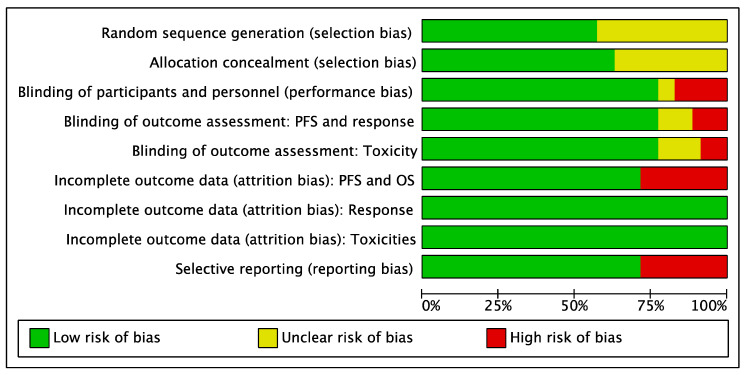
Risk of bias analysis.

The risk of bias analysis did not reveal a relevant risk of bias for five out of seven domains (Figure 6).

However, there was a 29% high risk of selective reporting bias/incomplete outcome-attrition bias, because 10 studies did not report OS outcome (Figure S3). Moreover, randomization and the methodology applied to assign patients to each treatment arm were not clear in 16 studies (47%), thus increasing the risk of selection bias (Figure S3).

## 4. Discussion

Based on our pooled results, we estimated that the novel ET ± TT introduced in the last 20 years, compared to the older endocrine therapeutic standards, produced a relative reduction in the risk of disease progression ranging from 41% to 26% and a relative reduction in the risk of death ranging from 27% to 12%, depending on the clinical subset. 

### 4.1. Postmenopausal, Visceral and Endocrine-Resistant Disease Subgroups

When we compared the different treatment strategies, combination regimens appeared to be favored over single agent ET in the postmenopausal, visceral and endocrine-resistant subgroups of patients. In fact, targeted agents are expected to boost the effect of single agent ET by reverting the molecular mechanisms of endocrine resistance [53]. They usually also provide an additional intrinsic antitumor effect. When further dissecting these results according to drug class, only the group of studies containing CDK4/6i-combinations provided significant individual pooled PFS and OS results versus the other treatment groups. Nevertheless, the OS differences observed in the three subgroups did not reach statistical significance, most likely due to a lack of statistical power. In fact, significant results were obtained in almost all subgroups when all results were pooled.

In the endocrine-resistant subset, the best result was obtained in the mTORi-containing group, which was also favored over the CDK4/6i group at the meta-regression. This was not completely unexpected given the degree of benefit (60% reduction in the risk of progression) obtained with everolimus + exemestane in the BOLERO2 trial in a context of substantial endocrine resistance [2]. In fact, many pathways of resistance converge on mTOR [54]. In addition, the sensitivity to CDK4/6i requires an intact Cyclin D1/Rb/E2F axis, and many of the signaling pathways that jeopardize sensitivity to endocrine therapy also lead to CDK4/6i resistance [55]. However, concerns have arisen over a potentially significant under powerfulness of the exemestane arm of the BOLERO 2 trial, due to the post-hoc finding of ESR1 mutations that might have prevented exemestane from producing any benefit, whereas a PFS improvement was observed with everolimus irrespective of patients’ mutational status [56]. Moreover, the CDK4/6i-containing group was the only group showing an individually significant pooled OS result in this subset, concordantly with what is observed in a recently published meta-analysis on CDK4/6i OS benefit [11]. Our result was mostly driven by the MONARCH-2 abemaciclib-containing study, similar to what occurs in the setting of primary endocrine resistance. In case of secondary endocrine resistance, the overall reduction in the risk of progression of around 40% was driven by everolimus-, alpelisib- (in PIK3CA-mutant patients) and abemaciclib-containing combinations. This suggests that abemaciclib might play a relevant role in the treatment of endocrine-resistant tumors. Unfortunately, our results of primary and secondary endocrine resistance should be taken with caution, due to the very limited number of studies with available results for these subgroups. Finally, positive individual PFS results in the AKTi- and HDACi-containing groups suggest that these drug classes might play a role in early treatment lines of endocrine-resistant disease. However, negative results for the E2112 phase III trial of entinostat + exemestane vs. exemestane in AI resistant HR+/HER2− MBC have been recently presented [57]. Hence, the future of HDACi in this clinical setting is uncertain. Conversely, the phase III CAPItello-291 trial is currently recruiting patients to confirm the efficacy of capivasertib + fulvestrant combination in AI-resistant HR+/HER2− MBC (ClinicalTrials.gov Identifier: NCT 04305496).

### 4.2. Bone-Only, Endocrine-Sensitive and Non-Visceral Disease Subgroups

Notably, ET + TT was more effective in bone-only disease than ET alone, despite the fact that this subgroup of tumors is usually considered an indolent, slow-growing, prognostically better entity than the other clinical subgroups of breast cancer [58,59], making it more difficult, in principle, to identify an additional beneficial effect from combination strategies. This result was driven by CDK4/6i studies, because it was the only group in which there was a significant and profound PFS benefit. This result is also concordant both with a recent meta-analysis conducted in the same subset testing multiple types of ET [58], and with a recent US Food and Drug Administration meta-analysis on CDK4/6i [60]. However, a severe disproportion of RCT between single agent and combination treatment study groups (1 vs. 12) should be considered in this context. Moreover, the only study of the single agent group compared fulvestrant at a suboptimal dosage to an AI in the secondary endocrine resistance subset, with negative results. Therefore, we cannot formally exclude the possibility that single agent ET might be a valid option for bone-only disease.

In endocrine-sensitive disease, there was no difference between combination treatments and single agent endocrine therapy. However, it is important to highlight that both strategies improved PFS. The subgroup analysis according to drug class revealed that CDK4/6i-containing studies had both the greatest PFS benefit and the numerically highest OS benefit, although the subgroup analysis was not statistically significant for OS.

Similarly, treatment strategies did not differ significantly in non-visceral disease, perhaps due to the greater efficacy displayed by fulvestrant over anastrozole in the first-line FALCON phase III trial [48], which was the only study contributing to the single agent group. In the subgroup analysis according to drug class, CDK4/6i-containing studies, compared to the other classes, had the most powerful effect. This result was confirmed in the meta-regression analysis, although a comparable effect was observed with fulvestrant. In terms of OS, only CDK4/6i-based regimens retained a significant individual result, which suggests that CDK4/6i-based regimens are the best option also in patients with non-visceral disease, as also suggested elsewhere [11]. However, in endocrine-sensitive tumors without visceral involvement, fulvestrant might be a valuable first-line option for selected patients (e.g., patients with contraindications to CDK4/6i).

### 4.3. PIK3CA Mutational Status

In the PIK3CA wild-type setting, CDK4/6i drove the overall PFS benefit observed over ET, whereas in PIK3CA-mutant patients, PFS was significantly better in both PI3Ki- and CDKi-containing groups versus single-agent ET. Interestingly, partial results of the BYLieve trial have been recently reported, confirming the efficacy of PI3Ki + ET in PIK3CA-mutant patients, even if pre-treated with CDK4/6i [61]. At the same time, modest results have been recently observed with the PI3Ki taselisib in combination with fulvestrant in AI-pretreated HR+/HER2− MBC in the SANDPIPER phase III trial [62]. Overall, our results, put into context, indicate that the efficacy of CDK4/6i + ET is not affected by PIK3CA mutational status and can thus be used in both PIK3CA-mutant and wild-type patients, while PI3Ki + ET combinations might play a significant role in the early treatment of patients whose tumors harbor PIK3CA mutations and can be used after CDK4/6i-based combinations, without significant concerns regarding their efficacy. However, not all PI3Ki are the same and only alpelisib has thus far shown an adequate therapeutic efficacy associated to a sufficiently manageable toxicity profile.

### 4.4. Limitations and Strengths

Our study has several limitations. First, not being a network meta-analysis, we were unable to directly compare all drugs or combinations of drugs to one and another. Consequently, some degree of precision was lost. Moreover, we were not able to conduce separate in-depth analyses for first- and second-line, since the majority of studies included patients from both treatment lines and did not provide separate results. Additionally, our analysis did not include toxicity comparisons, as well as quality of life (QoL), being essentially focused on survival endpoints. Nevertheless, a previous work from our group comprehensively resumed main toxicities for all available single agent ET, CT and ET + TT combinations, including treatments analyzed in the present study [19]. Differently, QoL is difficult to evaluate in a meta-analyses such as ours that include numerous studies involving a high number of different drugs/regimens. In any case, although QoL was not assessed and/or not reported for 22 (62.9%) of the included RCT, we briefly resumed available results in Table S1. No differences between experimental and standard arms were the most frequently reported outcome and CDK4/6i-based combinations were the ones most frequently ameliorating patients’ QoL, compared to standard treatment arm.

We were not able to perform an individual patient data (IPD) meta-analysis, due to the lack of the necessary resources. In any case, it is worth mentioning that for meta-analyses of published time-to-event outcomes as ours, individual case studies have shown that they can produce effects that are larger than, smaller than, or similar to their IPD equivalents [63]. Moreover, hazard ratios from published aggregate patients’ data (APD) meta-analyses seem to most likely agree with those from IPD, when the information size is large [64].

Finally, we assessed the presence of publication bias through funnel plots inspection and Egger’s test (when applicable), whose use and interpretation have been controversial because of concerns about statistical validity, appropriate interpretation and low power of the tests [65,66]. However, they still represent the standard recommended and most easy-to-interpret techniques [67]. In any case, the presence of publication bias was only observed for the PFS postmenopausal pooled result, where this outcome was not completely unexpected, due to a potential “selective analysis reporting” [68]. In fact, for the scope of our study, we excluded all RCT regarding endocrine agents and combinations not used in clinical practice over the last 20 years or not sufficiently promising to be expected to be introduced into the clinical scenario within a reasonable time. Most of these drugs were investigated precisely in the postmenopausal setting.

To our knowledge, this analysis is the first to produce a comprehensive and contextualized evaluation of the impact on disease progression delay and survival increase of endocrine-based treatments developed in the last 20 years, which specifically focused on specific disease settings of clinical relevance. In this view, strengthening the efficacy of ET ± TT, especially in the visceral and endocrine resistant subgroups, two controversial subsets when considering the issue of the overuse of upfront CT [69,70,71,72], is of outmost importance to ultimately provide patients with the most efficacious treatment options. Despite numerous therapeutic advances, we were also able to identify a potentially tailored role for upfront single agent ET in the era of TT-based combinations. Heterogeneity was properly assessed by adopting a random-effect model, with subgroup analyses and metaregressions. Finally, the risk of bias analysis did not raise substantial concerns regarding the internal validity of the studies included.

## 5. Conclusions

Combination strategies appear to be more beneficial than single agent ET in the treatment of postmenopausal, visceral, bone-only and endocrine-resistant tumors, while single agent ET might still be considered in selected cases for the upfront treatment of endocrine-sensitive tumors and in tumors without metastatic visceral involvement. CDK4/6i+ET combinations were the most effective treatment in the first-/second-line settings irrespective of tumor metastatic distribution and PIK3CA mutational status, as well as in endocrine-sensitive tumors. In the case of endocrine-resistance, CDK4/6i-based regimens were significantly and consistently effective in prolonging both PFS and OS, however mTORi-based regimens were apparently favored over the others, although some concern remains regarding this result. PI3Ki + ET regimens were effective in PIK3CA-mutant patients. Overall, we provide strong evidence to further support the main international treatment guidelines [16,17,18] and to help clinicians in tailoring their treatment choices in specific patients’ subgroups.

## Figures and Tables

**Table 1:** PFS and OS pooled results according to each patients’ subgroup.

PFS/TTP						
Subset	N. Comparisons ^b^	Pooled HR (95% CI)	I^2^ (%)	*p*_pooled_	*p*_heterogeneity_	Publication Bias
						(Egger’s Test *p*)
Pre/perimenopausal	4	0.59 (0.45–0.76)	46.09%	<0.001	0.121	NE
Postmenopausal ^a^	32	0.70 (0.63–0.78)	86.2%	<0.001	<0.001	0.009
Visceral	19	0.68 (0.60–0.77)	66.50%	<0.001	<0.001	0.47
Non-visceral	14	0.64 (0.57–0.72)	22.90%	<0.001	0.21	0.74
Bone-only	13	0.67 (0.53–0.84)	56.50%	<0.001	0.006	0.81
Endocrine Sensitive	18	0.62 (0.51–0.75)	85.40%	<0.001	<0.001	0.15
Endocrine Resistant	19	0.71 (0.62–0.82)	82.10%	<0.001	<0.001	0.09
Primary Resistance	4	0.63 (0.46–0.86)	30.90%	0.004	0.23	NE
Secondary Resistance	4	0.60 (0.51–0.71)	0.00%	<0.001	0.75	NE
PIK3CA-mutant	5	0.59 (0.50–0.70)	0.00%	<0.001	0.57	NE
PIK3CA-wild-type	5	0.74 (0.55–0.99)	70.90%	0.042	<0.001	NE
**OS**						
Pre/perimenopausal	3	0.76 (0.60–0.96)	0.00%	0.02	0.41	NE
Postmenopausal ^a^	21	0.85 (0.77–0.94)	55.0%	<0.001	0.003	0.47
Visceral	6	0.80 (0.71–0.91)	0.00%	<0.001	0.77	NE
Non-visceral	5	0.73 (0.62–0.87)	0.00%	<0.001	0.76	NE
Bone-only	4	0.74 (0.58–0.94)	0.00%	0.01	0.44	NE
Endocrine Sensitive	9	0.80 (0.68–0.93)	59.9%	0.005	0.01	0.44
Endocrine Resistant	11	0.88 (0.81–0.95)	26.7%	0.002	0.19	0.35
PIK3CA-mutant	2	0.80 (0.59–1.03)	0.00%	0.08	0.76	NE
PIK3CA-wild-type	2	0.99 (0.79–1.24)	34.70%	0.94	0.21	NE

PFS, progression-free survival; TTP, time-to-progression; OS, overall survival; HR, hazard ratio; 95% CI, 95% confidence interval; NE: not estimable. ^a^ Postmenopausal is here considered as only physiological or due to surgical castration before study enrollment. ^b^ The number of comparisons can be superior to the number of studies.

**Table 2:** The results of PFS/TTP subgroup analyses and meta-regressions for treatment categories and drug classes.

Subsets	Comparisons	N. Comparisons	Pooled HR	*p*_subgroup_	I^2^ (%)	*β*_meta-regression_	*p*_meta-regression_
Postmenopausal ^a^	ET vs. ET	13	0.82 (0.69–0.97)	0.01	87.9%	Reference	0.01
	ET + TTorComb. vs. ET	19	0.63 (0.56–0.71)		78.8%	−0.16	
Postmenopausal ^a^	CDK4/6i + ET vs. ET	7	0.55 (0.50–0.61)	0.01	0.0%	Reference	-
	AI vs. Progestins	1	0.99 (0.79–1.24)		NE	0.60	0.04
	AI vs. SERM	3	0.44 (0.21–0.92)		96.2%	−0.08	0.70
	AKTi + ET vs. ET	1	0.58 (0.40–0.85)		NE	0.07	0.84
	HDACi + ET vs. ET	1	0.73 (0.50–1.07)		NE	0.30	0.38
	mTORi + ET vs. ET	4	0.58 (0.36–0.94)		92.1%	0.07	0.68
	PI3Ki + ET vs. ET	4	0.73 (0.66–0.81)		0.0%	0.27	0.13
	SERD + AI vs. AI	1	0.80 (0.68–0.94)		NE	0.39	0.17
	SERD + AI vs. SERD	1	1.00 (0.83–1.21)		NE	0.61	0.03
	SERD vs. AI/SERM	8	0.96 (0.85–1.08)		55.0%	0.56	<0.001
Visceral	ET vs. ET	2	0.95 (0.79–1.15)	<0.001	0.00%		0.028
	ET+TT or ET comb. vs. ET	17	0.65 (0.57–0.74)		60.70%	−0.30	
Visceral	CDK4/6i + ET vs. ET	8	0.55 (0.49–0.61)	<0.001	0.00%	Reference	
	HDACi + ET vs. ET	1	0.76 (0.47–1.22)		NE	0.36	0.19
	PI3Ki + ET vs. ET	4	0.67 (0.57–0.79)		16.00%	0.21	0.02
	SERD + AI vs. AI	1	0.79 (0.63–0.99)		NE	0.36	0.006
	SERD + AI vs. SERD	1	1.10 (0.87–1.40)		NE	0.69	0.001
	SERD vs. AI/SERM	2	0.95 (0.79–1.15)		0.00%	0.55	0.001
Non-visceral	ET vs. ET	1	0.59 (0.42–0.84)	0.63	NE	Reference	
	ET+TT or ET comb. vs. ET	13	0.65 (0.57–0.74)		27.90%	0.09	0.69
Non-visceral	CDK4/6i + ET vs. ET	6	0.55 (0.47–0.63)	0.008	0.00%	Reference	
	PI3Ki + ET vs. ET	4	0.78 (0.64–0.95)		0.00%	0.36	0.004
	SERD + AI vs. AI	1	0.84 (0.61–1.15)		NE	0.43	0.01
	SERD vs. AI/SERM	1	0.59 (0.42–0.84)		NE	0.08	0.67
Bone-only	ET vs. ET	1	1.37 (0.83–2.26)	0.004	NE	Reference	
	ET+TT or ET comb. vs. ET	12	0.62 (0.50–0.77)		41.80%	−0.79	0.03
Bone-only	CDK4/6i + ET vs. ET	7	0.50 (0.40–0.62)	0.002	10.70%	Reference	
	HDACi + ET vs. ET	1	0.89 (0.37–2.12)		NE	0.58	0.21
	PI3Ki + ET vs. ET	2	0.79 (0.47–1.34)		17.70%	0.46	0.09
	SERD +AI vs. AI	1	0.77 (0.54–1.10)		NE	0.44	0.07
	SERD+AI vs. SERD	1	0.99 (0.61–1.60)		NE	0.69	0.018
	SERD vs. AI/SERM	1	1.37 (0.83–2.56)		NE	1.01	0.001
Endocrine Sensitive	ET vs. ET	5	0.59 (0.37–0.94)	0.79	94.90%	Reference	
	ET + TT or ET comb. vs. ET	13	0.63 (0.53–0.74)		59.80%	0.039	0.85
Endocrine Sensitive	CDK4/6i + ET vs. ET	7	0.52 (0.46–0.59)	<0.001	0.00%	Reference	
	AI vs. SERM	2	0.31 (0.06–1.59)		97.8%	−0.43	0.21
	HDACi + ET vs. ET	1	0.85 (0.54–1.34)		NE	0.43	0.31
	mTORi + ET vs. ET	1	0.87 (0.69–1.10)		NE	0.51	0.24
	PI3Ki + ET vs. ET	1	0.87 (0.35–2.17)		NE	0.51	0.41
	SERD + AI vs. AI	1	0.80 (0.68–0.94)		NE	0.43	0.32
	SERD vs. AI/SERM	3	0.87 (0.62–1.22)		82.9%	0.50	0.08
Endocrine Resistance	ET vs. ET	6	0.92 (0.85–1.00)	<0.001	0.00%	Reference	
	ET + TT or ET comb. vs. ET	13	0.60 (0.50–0.72)		75.40%	−0.43	0.001
Endocrine Resistance	CDK4/6i + ET vs. ET	6	0.56 (0.49–0.65)	<0.001	0.00%	Reference	
	AKTi + ET vs. ET	1	0.58 (0.40–0.85)		NE	0.03	0.88
	AI vs. Progestins	1	0.99 (0.79–1.24)		NE	0.56	0.001
	HDACi + ET vs. ET	1	0.47 (0.23–0.97)		NE	−0.18	0.62
	mTORi + ET vs. ET	2	0.47 (0.29–0.74)		74.30%	−0.29	0.016
	PI3Ki + ET vs. ET	2	0.69 (0.57–0.84)		0.00%	0.2	0.092
	SERD + AI vs. SERD	1	1.00 (0.83–1.21)		NE	0.57	0.001
	SERD vs. AI/SERM	4	0.95 (0.87–1.05)		0.00%	0.53	0.001
PIK3CA-mutant	CDK4/6i + ET vs. ET	1	0.48 (0.30–0.77)	0.38	NE	NE	NE
	PI3Ki + ET vs. ET	4	0.60 (0.50–0.72)		0.00%		
PIK3CA-wild-type	CDK4/6i + ET vs. ET	1	0.45 (0.31–0.65)	0.001	NE	NE	NE
	PI3Ki + ET vs. ET	4	0.87 (0.72–1.03)		7.20%		

PFS, progression-free survival; TTP, time-to-progression; HR, hazard ratio; 95% CI, 95% confidence interval; ET, single agent classic endocrine therapy; ET comb., combination of classic endocrine therapies; i, inhibitor; AI, aromatase inhibitor; i, inhibitor; SERD, selective estrogen receptor degrader/downregulator (fulvestrant); SERM, selective estrogen receptor modulator (tamoxifen); HDAC, histone deacetylase; NE, not estimable. ^a^ Postmenopausal is here considered as only physiological or due to surgical castration before study enrollment.

## Data Availability

The databases for the analyses of this study are available on request from the corresponding author.

## Supplementary Materials

The following are available online at https://www.mdpi.com/2072-6694/13/6/1458/s1, Figure S1: Funnel plots, Figure S2: PFS according to drug class comparisons, Figure S3: Detailed risk of bias analysis for each study, Table S1: Characteristics of the included trials, Table S2: Overall survival analyses according to treatment category and drug class, Text S1: Supplementary Methods. References [73,74,75,76,77,78,79,80,81,82,83,84,85] are cited in the Supplementary Materials.
