# Supplementary material for: Endocrine-Based Treatments in Clinically-Relevant Subgroups of Hormone Receptor-Positive/HER2-Negative Metastatic Breast Cancer: Systematic Review and Meta-Analysis
Source: Cancers (Basel). 2021 Mar 22;13(6):1458. doi: 10.3390/cancers13061458 (PMC8004645; doi:10.3390/cancers13061458)
Supplement: Supplementary file 1 [file cancers-13-01458-s001.pdf]

Supplementary Material: Endocrine-Based Treatments in Clinically-Relevant Subgroups of Hormone Receptor-Positive/HER2-Negative Metastatic Breast Cancer: Systematic Review and Meta-Analysis

Francesco Schettini, Mario Giuliano, Fabiola Giudici, Benedetta Conte, Pietro De Placido, Sergio Venturini , Carla Rognoni, Angelo Di Leo, Mariavittoria Locci, Guy Jerusalem, Lucia Del Mastro, Fabio Puglisi, PierFranco Conte, Michelino De Laurentiis, Lajos Pusztai, Mothaffar F. Rimawi, Rachel Schiff, Grazia Arpino, Sabino De Placido, Aleix Prat and Daniele Generali

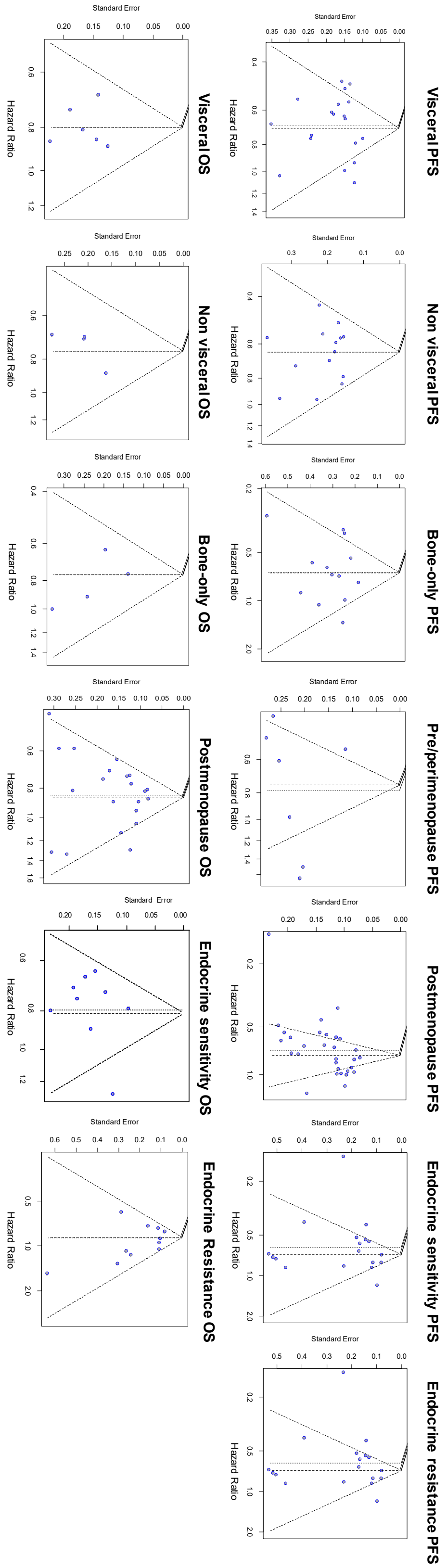

Figure S1. Funnel plots PFS, progression-free survival; OS, overall survival.

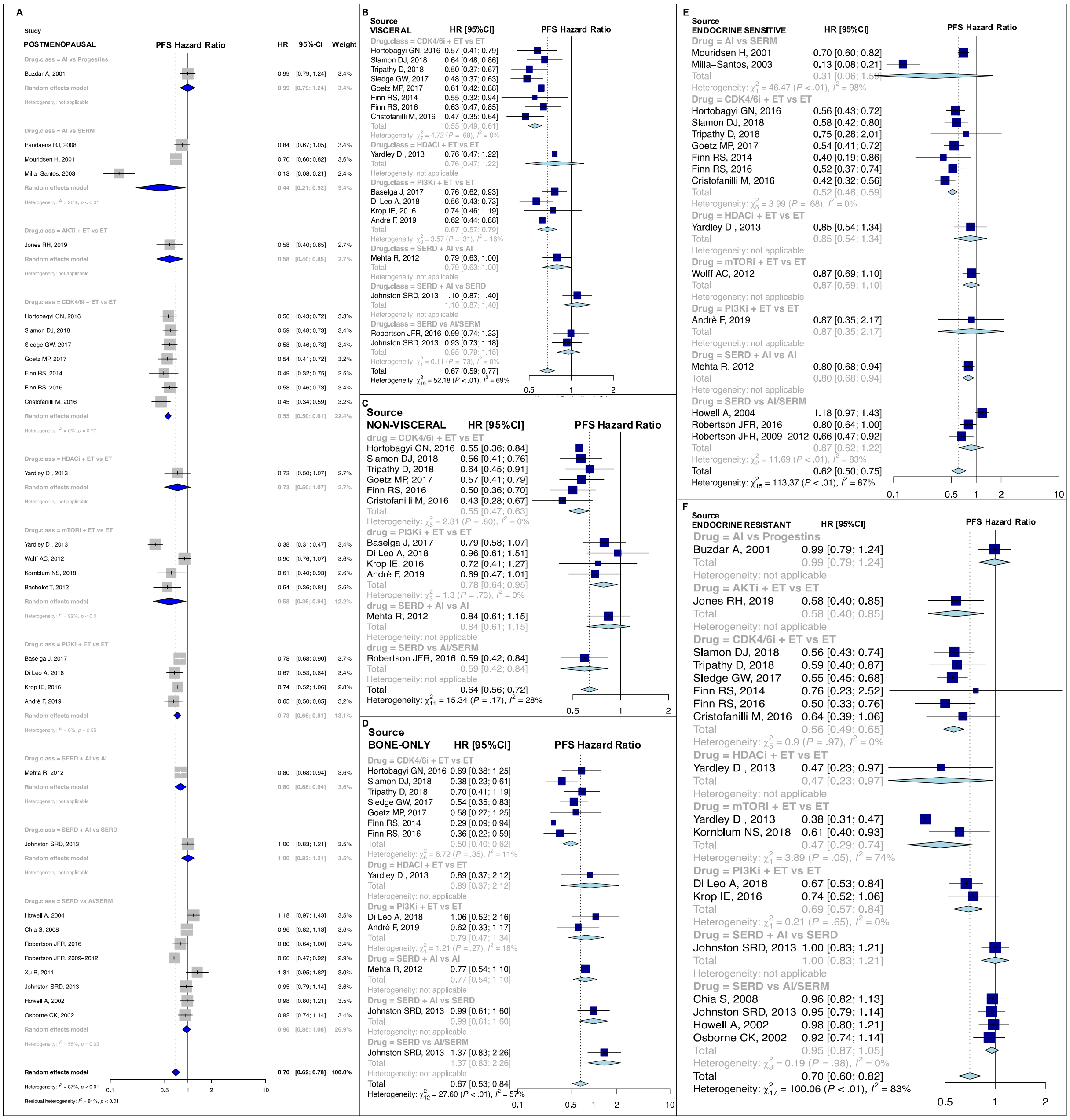

Figure S2. PFS according to drug class comparisons. PFS pooled results according to drug class comparisons in postmenopausal (**A**), visceral (**B**), non-visceral (**C**), bone-only (**D**), endocrine sensitive (**E**) and resistant (**F**) disease; PFS, progression-free survival, ET, single agent endocrine therapy; HR, hazard ratio; 95% CI, 95% confidence interval; PI3Ki, phosphatidylinositol 3-kinases inhibitors; CDK4/6i, Cyclin-dependent kinases 4/6 inhibitors; SERD, selective estrogen receptor downregulators; SERM, selective estrogen receptor modulators; AI, aromatase inhibitor; AKTi, AKT inhibitors; mTORi, mTOR inhibitors; HDACi, histone deacetylase inhibitors.

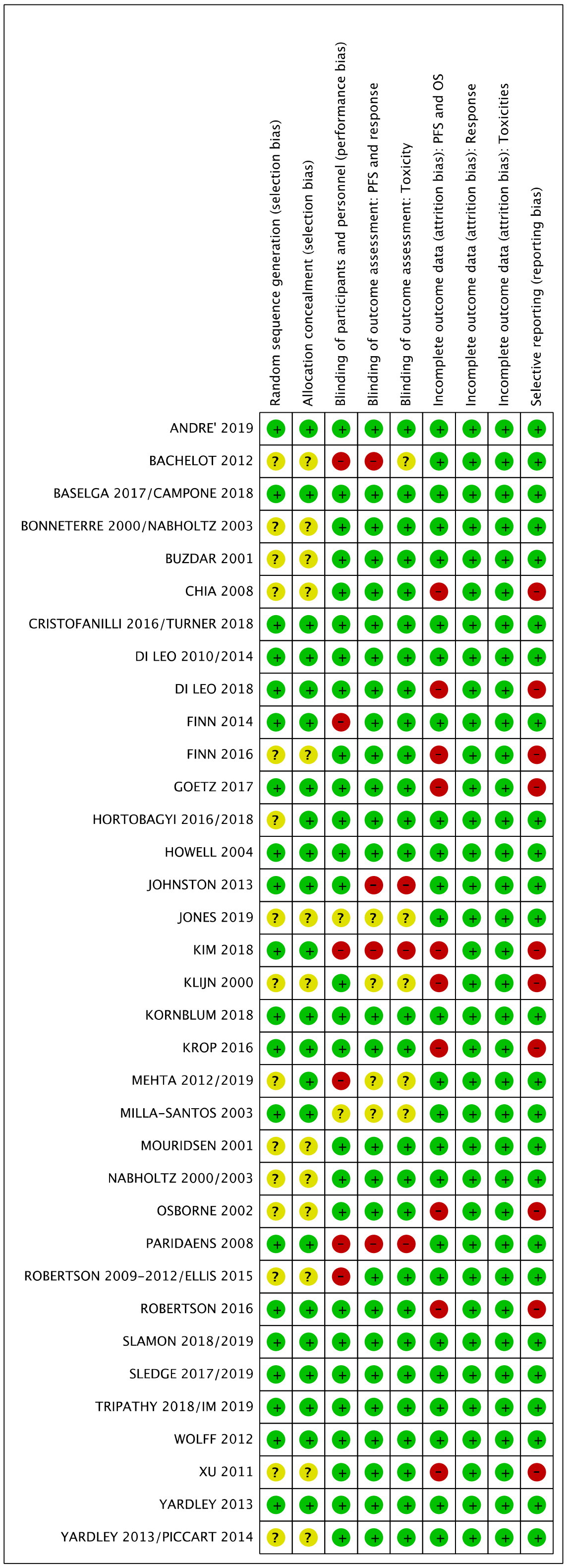

Figure S3. Detailed risk of bias analysis for each study. Green: low risk of bias; Yellow: unclear risk of bias; Red: high risk of bias.

Table S1. Characteristics of the included trials.

Trial	First author	Year	Journal	Phase	Line	Pts Number	Follow-Up	Centers	Endpoints	QoL Results*	Comparison	Study Category	Drug Class	Menopausal setting
-	Buzdar A.	2001	J. Clin. Oncol.	III	≥1	602	18	multicenter	PFS/TTP	NR	LET vs. MEG AC	Single agent ET comparison	AI vs. Progestins	post
-	Mouridsen H.	2001	J. Clin. Oncol.	III	1	916	32	multicenter	PFS/TTP	NR	LET vs. TAM	Single agent ET comparison	AI vs SERM	post
-	Milla-Santos	2003	Am. J. Clin. Oncol.	III	1	238	13.3	-	PFS/TTP; OS	NR	ANA vs. TAM	Single agent ET comparison	AI vs. SERM	post
-	Howell A.	2004	J. Clin. Oncol.	III	1	587	14.5	multicenter	PFS/TTP; OS	No difference	FULV HD vs. TAM	Single agent ET comparison	SERD vs. AI/SERM	post
BELLE 2	Baselga J./Campone M.	2017/2018	Lancet Oncol./Eur. J. Cancer	III	≥1	1147	4.2–5.0	multicenter	PFS/TTP; OS	NR	BUPA + FULV HD vs. FULV HD	ET+TT or ET combinations	PI3Ki + ET vs. ET	post
BELLE 3	Di Leo A.	2018	Lancet Oncol.	III	≥2	432	-	multicenter	PFS/TTP	NR	BUPA + FULV HD vs. FULV HD	ET+TT or ET combinations	PI3Ki + ET vs. ET	post
BOLERO 2	Yardley D./Piccart M./Campone M.	2013/2014/2013	Adv. Ther./Ann. Oncol./Curr. Med. Res. Opin.	III	≥1	724	7.6	multicenter	PFS/TTP; OS	No difference	EVE + EXE vs. EXE	ET+TT or ET combinations	mTORi + ET vs. ET	post
CONFIRM	Di Leo A.	2010/2014	J. Clin. Oncol./J. Natl. Cancer Inst.	III	≥1	736	-	multicenter	PFS/TTP; OS	No difference	FULV HD vs. FULV LD	Single agent ET comparison	-	post
EFECT	Chia S.	2008	J. Clin. Oncol.	III	1	540	13	multicenter	PFS/TTP	No difference	FULV ID vs. EXE	Single agent ET comparison	SERD vs. AI/SERM	post
FAKTION	Jones R.H.	2019	ASCO	II	≥1	170	12.4–11.8	multicenter	PFS/TTP; OS	NR	CAP + FULV HD vs. FULV HD	ET+TT or ET combinations	AKTi + ET vs. ET	post
FALCON	Robertson J.F.R.	2016	Lancet	III	1	462	25	multicenter	PFS/TTP	No difference	FULV HD vs. ANA	Single agent ET comparison	SERD vs. AI/SERM	post
FERGI	Krop I.E.	2016	Lancet Oncol.	III	≥1	168	6	multicenter	PFS/TTP	NR	PIC + FULV HD vs. FULV HD	ET+TT or ET combinations	PI3Ki + ET vs. ET	post
FIRST	Robertson J.F.R./Ellis M.J.	2009-2012/2015	J. Clin. Oncol./Breast Cancer Res. Treat./J. Clin. Oncol.	II	1	205	18.8–12.9	multicenter	PFS/TTP; OS	NR	FULV HD vs. ANA	Single agent ET comparison	SERD vs. AI/SERM	post
FLAG	Kim J.I.	2018	Eur. J. Cancer	II	≥1	138	32.2	multicenter	PFS/TTP	NR	FULV HD + LHRHA vs. LHRHA	ET+TT or ET combinations	-	pre
FLAG	-	-	-	-	-	-	-	-	PFS/TTP	NR	ANA + LHRHA vs. LHRHA	ET+TT or ET combinations	-	pre
HORIZON	Wolff A.C.	2012	J. Clin. Oncol.	III	1	1112	9.5	multicenter	PFS/TTP; OS	NR	TEM + LET vs. LET	ET+TT or ET combinations	mTORi + ET vs. ET	post
MONALEESA 2	Hortobagyi G.N./Verma S.	2016/2018/2018	N. Engl. J. Med./Ann. Oncol./Breast Cancer Res. Treat.	III	1	668	15.3	multicenter	PFS/TTP; OS	No difference	RIBO + LET vs. LET	ET+TT or ET combinations	CDK4/6i + ET vs. ET	post

Trial	First author	Year	Journal	Phase	Line	Pts Number	Follow-Up	Centers	Endpoints	QoL Results*	Comparison	Study Category	Drug Class	Menopausal setting
MON-ALEESA 3	Slamon D.J./Beck J.T.	2018/2019/2019	J. Clin. Oncol./N. Engl. J. Med./Cancer Res.	III	≥1	726	20.4	multicenter	PFS/TTP; OS	In favor of experimental	RIBO + FULV HD vs FULV HD	ET+TT or ET combinations	CDK4/6i + ET vs. ET	post
MON-ALEESA 7	Tripathy D./Seock-Ah I./Harbeck N.	2018/2019/2020	Lancet Oncol./N. Engl. J. Med./Ther. Adv. Med. Oncol.	III	1	672	19.2	multicenter	PFS/TTP; OS	In favor of experimental	RIBO + NSAI/TAM vs. NSAI/TAM	ET+TT or ET combinations	CDK4/6i + ET vs. ET	pre
MON-ARCH 2	Sledge G.W./Kaufman P.A.	2017/2019/2020	J. Clin. Oncol./JAMA Oncol./Oncologist	III	≥1	669	19.5	multicenter	PFS/TTP; OS	Mostly in favor of experimental	ABE + FULV HD vs. FULV HD	ET+TT or ET combinations	CDK4/6i + ET vs ET	pre/post
MON-ARCH 3	Goetz M.P.	2017/2020	J. Clin. Oncol./Oncologist	III	1	493	17.8	multicenter	PFS/TTP	No difference	ABE + ANA/LET vs. ANA/LET	ET+TT or ET combinations	CDK4/6i + ET vs. ET	post
EN-CORE301	Yardley D.	2013	J. Clin. Oncol.	II	≥1	130	24–26.4	multicenter	PFS/TTP; OS	NR	ENT + EXE vs. EXE	ET+TT or ET combinations	HDACi + ET vs ET	post
North American Trial	Nabholtz J.M.	2000/2003	J. Clin. Oncol.	III	1	353	17.7	multicenter	PFS/TTP; OS	NR	ANA vs. TAM	Single agent ET comparison	AI vs. SERM	post
PALOMA 1	Finn R.S.	2014	Lancet Oncol.	II	1	165	29.6–27.9	multicenter	PFS/TTP; OS	NR	PALBO + LET vs. LET	ET+TT or ET combinations	CDK4/6i + ET vs. ET	post
PALOMA 2	Finn R.S./Rugo H.S.	2016/2018	N. Engl. J. Med./Ann. Oncol.	III	1	666	23	multicenter	PFS/TTP	Slightly in favor of experimental	PALBO + LET vs. LET	ET+TT or ET combinations	CDK4/6i + ET vs. ET	post
PALOMA 3	Cristofanilli M./Turner N.C./Harbeck N.	2016/2018/2016	Lancet Oncol./N. Engl. J. Med./Ann. Oncol.	III	≥1	521	8.9	multicenter	PFS/TTP; OS	In favor of experimental	PALBO + FULV HD vs. FULV HD	ET+TT or ET combinations	CDK4/6i + ET vs. ET	pre/post
PrECOG 0102	Kornblum N.S.	2018	J. Clin. Oncol.	II	≥1	131	19.3	multicenter	PFS/TTP; OS	NR	EVE + FULV HD vs. FULV HD	ET+TT or ET combinations	mTORi + ET vs. ET	post
SoFEA	Johnston S.R.D.	2013	Lancet Oncol.	III	≥1	723	37.9	multicenter	PFS/TTP; OS	NR	FULV ID + ANA vs. FULV HD	ET+TT or ET combinations	SERD + AI vs. SERD	post
SoFEA	-	-	-	-	-	-	-	-	PFS/TTP; OS	NR	FULV ID vs. EXE	Single agent ET comparison	SERD vs. AI/SERM	post
SOLAR 1	Andrè F.	2019	N. Engl. J. Med.	III	≥1	572	-	multicenter	PFS/TTP	No difference	ALP + FULV HD vs. FULV HD	ET+TT or ET combinations	PI3Ki + ET vs. ET	post
SWOG trial	Mehta R.	2012/2019	N. Engl. J. Med.	III	1	694	35	multicenter	PFS/TTP; OS	NR	FULV ID + ANA vs. ANA	ET+TT or ET combinations	SERD + AI vs. AI	post
TAMRAD	Bachelot T.	2012	J. Clin. Oncol.	II	≥1	111	23.7–24.2	multicenter	PFS/TTP; OS	NR	EVE + TAM vs TAM	ET+TT or ET combinations	mTORi + ET vs. ET	post
TARGET	Bonneterre J./Nahboltz J.M.	2000/2003	J. Clin. Oncol.	III	1	668	19	multicenter	PFS/TTP; OS	NR	ANA vs. TAM	Single agent ET comparison	AI vs. SERM	post
Trial 0020	Howell A.	2002	J. Clin. Oncol.	III	≥1	451	14.4	multicenter	PFS/TTP	No difference	FULV LD vs. ANA	Single agent ET comparison	SERD vs. AI/SERM	post

Trial	First author	Year	Journal	Phase	Line	Pts Number	Follow-Up	Centers	Endpoints	QoL Results*	Comparison	Study Category	Drug Class	Menopausal setting
Trial 0021	Osborne C.K.	2002	J. Clin. Oncol.	III	≥1	400	16.8	multicenter	PFS/TTP	No difference	FULV LD vs. ANA	Single agent ET comparison	SERD vs. AI/SERM	post
-	Paridaens R.J.	2008	J. Clin. Oncol.	II/III	1	371	29	multicenter	PFS/TTP; OS	NR	EXE vs. TAM	Single agent ET comparison	AI vs. SERM	post
-	Xu B.	2011	Cancer Chemother. Pharmacol.	III	≥1	234	-	multicenter	PFS/TTP	NR	FULV LD vs. ANA	Single agent ET comparison	SERD vs. AI	post

PFS,progression-free survival; TTP, time-to-progression; OS, overall survival; LET, letrozole; ANA, anastrozole; EXE, exemestane; TAM, tamoxifen; FULV, fulvestrant; LHRHA, LHRH analogue; PALBO, palbociclib; RIBO, ribociclib; ABE, abemaciclib; ENT, entinostat; AL alpelisib; BUPA, buparlisib; PIC, pictilisib; EVE, everolimus; TEM, temsirolimus; CAP, capivasertib; ID, fulvestrant induction dose 500 mg, then 250 mg every 4 weeks; HD, fulvestrant high dose/current treatment schedule; LD, fulvestrant low dose of 250 mg without induction; AI, aromatase inhibitor; i, inhibitor; SERD, selective estrogen receptor degrader/downregulator (fulvestrant); SERM, selective estrogen receptor modulator (tamoxifen); HDAC, histone deacetylase; ET, single agent classic endocrine therapy; QoL, quality of life; NR, not reported in a full paper publication; * not referred to differences in toxicity rates, but on specific QoL assessment tool (e.g. internationally validated questionnaires).

Table S2. Overall survival analyses according to treatment category and drug class.

Subsets	Comparisons	N. Comparisons	Pooled HR	$p_{subgroup}$	I^2 (%)	$\beta_{metaregression}$	$p_{metaregression}$
Postmenopausal	ET vs. ET	6	0.98 (0.77–1.24)	0.03	74.1%	Reference	
	ET + TTorComb. Vs. ET	14	0.82 (0.75–0.88)		10.8%	−0.16	0.02
Postmenopausal	CDK4/6i + ET vs. ET	5	0.75 (0.66–0.85)	0.20	0.0%	Reference	
	AI vs. AI	1	1.33 (0.78–2.26)		-	0.57	0.09
	AI vs. SERM	2	0.85 (0.49–1.49)		86.2%	0.13	0.49
	AKTi + ET vs. ET	1	0.59 (0.34–1.04)		-	−0.24	0.49
	HDACi + ET vs. ET	1	0.59 (0.36-0.97)		-	−0.24	0.45
	mTORi + ET vs. ET	4	0.85 (0.65–1.13)		53.0%	0.13	0.43
	PI3Ki + ET vs. ET	1	0.87 (0.74–1.02)		-	0.15	0.49
	SERD +AI vs. AI	1	0.82 (0.69- 0.98)		-	0.09	0.67
	SERD vs. AI/SERM	3	1.00 (0.74–1.36)		76.1%	0.24	0.30
	SERD + AI vs. SERD	1	0.95 (0.77–1.18)		-	0.30	0.06
Visceral	ET vs. ET	1	0.86 (0.56–1.33)	0.73	NE	NE	NE
	ET + TT or ET comb. Vs. ET	5	0.79 (0.70–0.90)		NE		
Visceral	CDK4/6i + ET vs. ET	4	0.76 (0.65–0.89)	0.59	0.00%	NE	NE
	SERD +AI vs. AI	1	0.88 (0.69–1.13)		NE		
	SERD vs. AI/SERM	1	0.86 (0.56–1.33)		NE		
No Visceral	ET vs. ET	1	0.68 (0.40–1.17)	0.77	-	NE	NE
	ET + TT or ET comb. vs. ET	4	0.74 (0.61–0.89)		0.00%		
No visceral	CDK4/6i + ET vs. ET	3	0.68 (0.54–0.85)	0.41	0.00%	NE	NE
	SERD + AI vs. AI	1	0.88 (0.64–1.21)		-		
	SERD vs. AI/SERM	1	0.68 (0.40–1.17)		-		
Bone-only	CDK4/6i + ET vs. ET	3	0.82 (0.60–1.13)	0.30	0.00%	NE	NE
	SERD +AI vs. AI	1	0.63 (0.43–0.93)		-		
Endocrine-Sensitive	ET vs. ET	3	0.82 (0.51–1.34)	0.81	88.0%	NE	NE
	ET+TTorComb. Vs. ET	6	0.78 (0.69–0.87)		-		
Endocrine-Sensitive	AI vs. SERM	1	0.64 (0.47–0.87)	0.57	-	NE	NE
	CDK4/6i + ET vs. ET	4	0.73 (0.62–0.87)		0.00%		
	SERD vs. AI/SERM	2	0.93 (0.48–1.80)		90.1%		
	mTORi + ET vs. ET	1	0.89 (0.65–1.22)		-		
	SERD +AI vs. AI	1	0.79 (0.65–0.96)		-		
Endocrine-Resistant	ET vs. ET	2	0.91 (0.70–1.18)	0.81	74.6%	NE	NE
	ET+TTorComb. Vs. ET	9	0.87 (0.77–0.99)		17.4%		
Endocrine-Resistant	CDK4/6i + ET vs. ET	4	0.80 (0.68–0.95)	0.33	19.3%	NE	NE
	mTORi + ET vs. ET	2	0.93 (0.76–1.12)		30.4%		
	AKTi + ET vs. ET	1	0.59 (0.34–1.04)		-		
	SERD vs. AI/SERM	1	1.05 (0.85–1.30)		-		
	SERD +AI vs. AI	1	1.08 (0.64–1.81)		-		
	SERD +AI vs. SERD	1	0.95 (0.77–1.18)		-		

OS, overall survival; HR, hazard ratio; 95% CI: 95% confidence interval; ET, single agent classic endocrine therapy; ET comb., combination of classic endocrine therapies; i, inhibitor; AI, aromatase inhibitor; SERD, selective estrogen receptor degrader/down-regulator (fulvestrant); SERM, selective estrogen receptor modulator (tamoxifen); PI3Ki, phosphatidylinositol 3-kinases inhibitors; CDK4/6i, Cyclin-dependent kinases 4/6 inhibitors; ET vs. ET, studies comparing single endocrine agents therapies; ET + TTorComb. vs. ET, studies comparing endocrine therapies + target therapies or endocrine therapies combinations against single agent endocrine treatments; SERD, selective estrogen receptor downregolators; SERM, selective estrogen receptor modulators; AI, aromatase inhibitor; AKTi, AKT inhibitors; mTORi, mTOR inhibitors; HDACi, histone deacetylase inhibitors; NE, not estimable.

Text S1: Supplementary Methods

1. Literature Search Strategy

We used the systematic literature search that we performed in our previous study i.e., Giuliano et al. [19]. The following search terms were used: breast, mammary, cancer, neoplasm, oncology, tumor, malignancy, carcinoma, adenocarcinoma, sarcoma, metastasis, metastatic, advanced, secondary, recurrent, inoperable, disseminated, incurable, trial, study, randomized, randomized, randomly, first line, second line, first-line, second-line, chemotherapy, endocrine therapy, everolimus, afinitor, sdz-rad, rad001, 159351-69-6, cyclophosphamide, methotrexate, fluorouracil, 5FU, 5-FU, doxorubicin, mitoxantrone, epirubicin, paclitaxel, docetaxel, liposomal doxorubicin, nab-paclitaxel, nab paclitaxel, pegylated, eribulin, capecitabine, vinorelbine, carboplatin, cisplatin, platinum, gemcitabine, anastrozole, letrozole, aromatase inhibitor, exemestane, tamoxifen, palbociclib, PD-0332991, PD0332991, buparlisib, pictilisib, pi3k inhibitor, fulvestrant, faslodex, without language limitations. The following online archives were interrogated: Pubmed®, EMBASE®, Cochrane Central Register of Clinical Trials and Web of Science, American Society of Clinical Oncology (ASCO), European Society of Medical Oncology (ESMO) annual meetings, and San Antonio Breast Cancer Symposiums (SABCS). Reference lists from the most recent international guidelines, including the American National Comprehensive Cancer Network (NCCN), ESMO, ASCO and Italian Association of Medical Oncology (AIOM) and cross-references from published trials and reviews or meta-analyses concerning therapeutic strategies in HR+ MBC were used to identify additional trials. The most recent and complete RCTs were included in case of duplicate publication. Phase II or III RCTs published in the form of full paper or abstracts between 1 January 2000 and 31 December 2018 were included in the analysis. Abstracts were used only when full papers had not been published and we excluded RCTs of chemotherapy ± target therapies and studies comparing endocrine therapy with chemotherapy, which were not pertinent to this meta-analysis. We also added all endocrine therapy RCTs that were published in 2018 and 2019, or that were presented at ASCO/ESMO/SABCS congresses in the form of an abstract, and added the most recent overall survival results for included trials, that were published after the systematic literature search (see references in the paper [2–10,26–52,68,73–85]). Two reviewers (Francesco Schettini and Carla Rognoni) independently evaluated whether each selected RCT fulfilled the predetermined criteria in the original review process; additionally, for the present study, other two reviewers (Benedetta Conte and Pietro De Placido) helped revising the literature and conferences' proceedings for novel published studies. Another reviewer (Daniele Generali) was ultimately consulted in case of controversy.

2. Additional Methods

Progression-free survival (PFS) was defined as the time from randomization until disease progression or death; time-to-progression (TTP) was defined as the time from randomization until objective tumor progression, not including deaths; overall survival (OS) was defined as the time from randomization until death from any cause.

According to ESO-ESMO breast cancer guidelines [18], endocrine sensitivity was defined as the condition of de novo disease or disease relapsed after more than one year from the completion of adjuvant ET. Primary endocrine resistance was defined as (i) a relapse of the disease while on the first 2 years of adjuvant ET, or (ii) progression within the first 6 months of first line ET for MBC while on ET. Secondary endocrine resistance was defined as (i) a relapse while on adjuvant ET but after the first 2 years or within 12 months of completing adjuvant ET, or (ii) progression after 6 months from the start of ET in MBC while on ET.

Heterogeneity was considered very low in case of I^2 values under 25%, low for values between 25–50%, moderate for values between 50–75%, and high for values exceeding 75% [67].

Each domain related to a risk of bias was assessed in each trial because of evidence that risk of bias is associated with biased estimates of treatment effect. The domains were the following: random sequence generation; allocation concealment; blinding of participants and personnel; blinding of outcome assessment; incomplete outcome data; selective reporting; other bias. The authors' judgments were categorized as "low risk", "high risk" or "unclear risk" of bias, according to the criteria outlined in the Cochrane Handbook for Systematic Reviews of Interventions and the Cochrane Collaboration's Risk of Bias tool in Review Manager (see Section 2.4. Data Analysis for references).
